# Assessing the role of *Piscine orthoreovirus* in disease and the associated risk for wild Pacific salmon

**DOI:** 10.1186/s12915-023-01548-8

**Published:** 2023-05-19

**Authors:** Gideon Mordecai, Arthur L. Bass, Rick Routledge, Emiliano Di Cicco, Amy Teffer, Christoph Deeg, Andrew W. Bateman, Kristina M. Miller

**Affiliations:** 1 Institute for the Oceans and Fisheries, University of British Columbia, Vancouver, BC Canada; 2 Pacific Salmon Ecology and Conservation Laboratory, Department of Forest and Conservation Sciences University of British Columbia, Vancouver, BC Canada; 3 Pacific Biological Station, Fisheries and Oceans Canada, Nanaimo, BC Canada; 4 Department of Statistics and Actuarial Science, Simon Fraser University, Burnaby, BC Canada; 5 Pacific Salmon Foundation, Vancouver, BC Canada; 6 Department of Environmental Conservation, University of Massachusetts Amherst, Amherst, MA 01003 USA; 7 Department of Forest and Conservation Sciences, University of British Columbia, Vancouver, BC Canada; 8 Department of Ecology and Evolutionary Biology, University of Toronto, Toronto, ON Canada

**Keywords:** Salmon, Piscine orthoreovirus (PRV), Infectious hematopoietic necrosis virus (IHNV), Comparitive pathology, Respiratory performance, Disease ecology

## Abstract

This paper is a response to Polinski, M. P. et al. *Innate antiviral defense demonstrates high energetic efficiency in a bony fish*. BMC Biology **19**, 138 (2021). 10.1186/s12915-021-01069-2

**Supplementary Information:**

The online version contains supplementary material available at 10.1186/s12915-023-01548-8.

## Background

In a laboratory study on sockeye salmon, Polinski et al. [1] investigated a suite of transcriptional, metabolic, and histopathological responses to separate experimental challenges with infectious hematopoietic necrosis virus (IHNV) and *Piscine orthoreovirus* (PRV). The stated aim of their study was to determine the metabolic costs of viral exposure and the ensuing immune response. To test their hypotheses relating to the energetic costs of viral infection, the authors used IHNV as a virus associated with acute infection and PRV as a virus associated with low virulence. Polinski et al. conclude that PRV exposure is of little consequence to sockeye salmon, but we argue that this study is not adequate to make such a conclusion. In some instances, the data may even suggest — in accordance with observations from other Pacific salmon [2–6] — that PRV might cause ecologically relevant disease in sockeye. Moreover, the authors’ approach and interpretations failed to acknowledge the limitations of studying a chronic disease in wild fish within a controlled laboratory environment, free from ecological complexities that likely interact with infection. We argue that some of the study results were misinterpreted, a number of specific claims were unjustified, and the conclusions are altogether too strident.

Polinski et al. start with the assumption that respiration is tightly tied to energy consumption and argued that infection-related metabolic costs should be evident as changes in an organism’s respiratory performance. To measure the respiratory state of individual fish, as a proxy for costs of infection, Polinski et al. used a suite of fifteen measurements, developed by some of the authors [7] and known as the “integrated respiratory assessment protocol” (IRAP). The authors inferred that minimal metabolic cost (according to IRAP metrics) of IHNV “resistance” and PRV “tolerance” indicated that innate responses to infection were not metabolically expensive. The paper reports a vast quantity of data on host-pathogen interactions at various biological resolutions (e.g., molecular, cellular, organismal). As with any experimental investigation, however, such challenging data can be analyzed and interpreted in a variety of ways, and the inferences drawn reflect choices made by investigators. Below we highlight elements of the experimental design and interpretation of the data that we argue tended to overlook apparent physiological costs, and we suggest that these are potentially ecologically meaningful.

Our comments are largely focused on the reported physiological response of sockeye to PRV exposure, rather than the transcriptomic data or the IHNV challenge. We group our commentary according to themes adapted from a recent framework for evaluating epidemiological research [8]. First, we reveal several analytical choices which underrepresented the true effect of PRV exposure and which raise questions over the study’s ability to detect an effect of viral exposure, should it exist. Next, we review the broader interpretation in light of the emerging evidence that PRV is associated with disease in Pacific salmon. Finally, we conclude with a holistic discussion that considers the possible ecological effects of the disease. Although each of the points we identify is seemingly minor, we suggest that cumulatively they show evidence of a physiological impact of high-load PRV infections. We conclude that the study by Polinski et al. does not rule out the potential etiological role of PRV infections — and therefore the associated population-level risk — in sockeye salmon.

## Results

Our data reanalysis has identified a number of discrepancies in the analysis and interpretation, which we argue undermine the claims of Polinski et al. that PRV infection has minimal impact on sockeye physiology. A more objective treatment of the data makes clear that a reasonable reader could draw different conclusions than those offered in the original paper — or that it is not possible to draw firm conclusions at all. Below, we outline several major concerns based on principles of sound experimental design and our best efforts to reproduce the originally reported statistical analyses.

### Potential effects of PRV are overlooked

PRV is implicated in similar disease pathways, all of which involve substantial lysis of infected red blood cells, across Pacific salmon species. In Chinook salmon, PRV is associated with jaundice/anemia [2] and the effects of PRV infection appear to be analogous in rainbow trout (*Oncorhynchus mykiss*), coho salmon (*Oncorhynchus kisutch*), and other Pacific salmonids [3, 4, 6, 9]. Polinski et al. investigated several metabolic measures that suggest a further analogous response to PRV infection in sockeye, but the results are downplayed or unreported (Additional file 1: Supplementary text 1). For example, the authors note that the data in their Fig. 1, S1, and S4 show significant PRV-induced changes in excess post-exercise oxygen consumption duration (EPOCdur), hematocrit (the proportional volume of erythrocytes in blood), and hemoglobin concentration but these changes are dismissed as being contingent on ‘individual-specific factors unidentified in this study’. In this statement, the authors cast aside the evidence that PRV may have an effect on the physiology of the host without adequate explanation or consideration. Our reanalysis of their data indicates a significant impact of PRV exposure on excess post-exercise oxygen consumption (EPOC; Additional file 1: Supplementary Table 1). Due to the omission of two data points from the control group, this result was not reported by Polinski et al. in their original article, an error they have since acknowledged in a correction article.

**Fig. 1:**
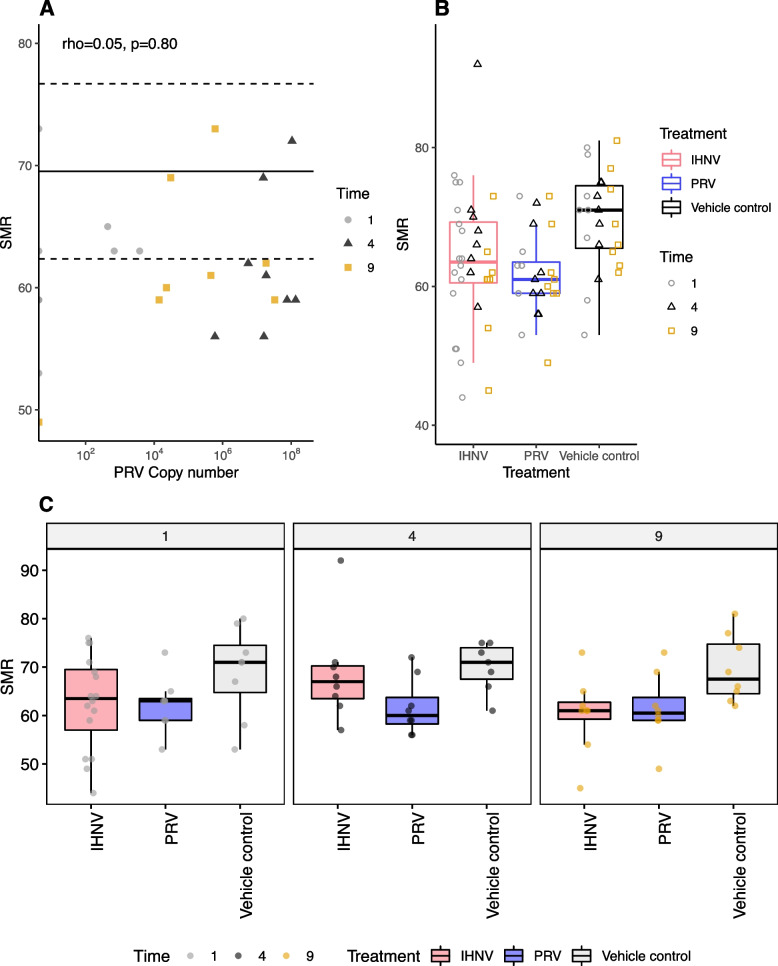
Standard metabolic rate is lower in PRV-challenged fish than in IHNV. **A** Correlation of SMR and PRV copy number. Horizontal solid line shows the mean of the control samples, dashed lines show the standard deviation from the mean. Points are shaped by time (weeks post challenge). The Spearman correlation and associated *p*-value are shown at the top of the plot. **B** When samples were pooled across time points, PRV-exposed fish have a lower overall SMR than the IHNV. Points are shaped by time point of samples (as in plot A). **C** At all time points, SMR for the PRV-exposed fish is consistently lower than the control group

In the case of standard metabolic rate (SMR), a statistical difference was observed between the control and the IHNV-exposed samples at week 9. However, the overall impact of PRV exposure was comparable to that of IHNV exposure (Fig. 1, also see Figure S1 in Polinski et al.). Lower SMR in PRV-exposed fish is evident at all time points (Fig. 1C), but not significant, likely attributable to the low power of the study design. These data were accurately displayed in their supplementary figures, but a reliance on statistical significance meant this impact was not noted or discussed. The authors found reduction of SMR in fish with lower IHNV loads but no effect on SMR in fish with high IHNV loads, and they suggest the low-load effect to be adaptive against hypoxia during systemic infection. The median SMR values were lower for PRV than for IHNV at all time points (Fig. 1), including at week 9, where IHNV exposure resulted in a statistically significant reduction in SMR (*p*=0.0457). We suggest that the “time specific” statistical analysis, small sample sizes, and an overreliance on statistical hypothesis testing [10], may well have resulted in underreporting of PRV’s effect.

Insufficient power (discussed in more detail below) makes real effects difficult to detect, and the sheer number of tests performed makes spurious results highly likely. For instance, using a significance threshold of 0.05, every 100 tests performed when the null hypothesis is true will yield about five “significant” false positive results. For their physiological and blood measures Polinski et al. performed 132 tests and reported 7 significant results using a 0.05 threshold, and while they adjusted p-values “for familywise error,” “no false discovery rate calculations were applied over the suite of comparisons.” It is thus virtually impossible to tell which “statistically significant” results reported by Polinski et al. are likely to be real and which ‘insignificant’ results are in fact evidence of biologically relevant processes. In this situation, biologically significant results — and not just technically statistically significant ones — should be discussed, especially when such results run counter to the authors’ assumptions or conclusions. Further study with a larger sample size is needed to determine the true effect of PRV on physiological stress, but the limited data available suggest that PRV-exposed fish may lower their metabolism to tolerate infection and protect against low oxygen, just as argued for low-load IHNV infections.

### Low statistical power and the inclusion of virus-negative fish in the viral treatment group

Statistical power (the probability of correctly rejecting a false null hypothesis) is directly influenced by a study’s sample size, and this affects the ability to answer the associated research questions. Although there are perfectly valid financial and practical limits to the scope and size of laboratory studies, the resultant power must still inform associated claims, especially where findings indicate a lack of statistical effect. Polinski et al. report that the power of their analyses was over 0.85. Their power analyses overlooked considerable relevant information, however. They only estimated power at large effect sizes [11], did not appear to consider that the focus of their analysis is on time-specific differences rather than the main effects of treatment, and did not seem to account for either Dunnett’s correction or complications due to non-independence of samples taken from the same tank. Together these oversights resulted in an overestimate — by up to an order of magnitude — of the study’s power. We have re-estimated the true power of the appropriate analyses to be in the range of 0.083 to 0.137 (Additional file 1: Supplementary Table 2, Supplementary text 2A). Our calculations still rely on the assumption of a “large effect size,” despite the fact that smaller effects would be even more difficult to detect but may remain biologically significant. We suggest that such an underpowered experimental design presents a very serious risk of falsely confirming any initial “no-effect” working hypotheses.

In addition to confounding the *a priori* power analysis, the issue of pseudoreplication also impacts the multiple comparisons. With only two tanks used for each viral treatment (with the exception of IHNV at week 1) and for the controls, it is effectively impossible to determine whether a given response variable is more impacted by unknown tank effects or the virus challenge itself. Polinski et al. treated individual fish as independent replicates, even if they came from the same tank, a classic example of pseudoreplication without an appropriate analytical framework [12]. Because the fish in each tank were subject to a common treatment, and unknown tank effects may be present (as they commonly are in such studies [13]), each fish is not a true independent replicate. We repeated the analysis but accounted for tank effects and found that fewer measures (four: EPOC at week 1 for both IHNV and PRV, non-IRAP hematocrit at week 1 for IHNV and at week 4 for PRV) were significantly different between treatment and control groups of fish (Additional file 1: Supplementary Table 1, Figure S2). It is not possible to determine whether this diminished result is due to inadequate statistical power or a true lack of any effects.

Additionally — and of critical importance — Polinski et al. did not differentiate viral exposure and infection in their study design, further contributing to the risk of inflated type II error (i.e. a false negative result). While the study was framed as an investigation of the energetic costs of viral response, individuals that were exposed to either virus, but that failed to show evidence of infection (via viral RT-qPCR screening) were included in the exposed group. Although these fish had technically been exposed to the virus, they were virus-negative and, in effect, uninfected. It is possible these fish fought off infection via an immune response. However, it seems more likely that the ability to resist infection is only short-lived since almost all fish are infected at later time points (we note that an author correction has since found that in fact all fish were infected at the two later time points, and the PRV negative fish were in fact false negative results). Regardless, to include the virus-negative fish from the first time point in the ‘infected’ group is misleading, since they dilute any effects of infection in the experimental group (Additional file 1: Supplementary text 2B, Figure S3). Had the study been framed as an investigation of PRV *exposure*, not PRV *infection*, this would have been more acceptable. Although we agree that the experimental setup was broadly suitable to examine the transcriptomic response to exposure, classifying the fish negative for the virus as “infected” throughout the main text of the manuscript was misleading. This is especially true given that PRV load was significantly correlated with several measures including hemoglobin, hematocrit, and EPOCdur (Additional file 1: Figure S1).

We carried out a comparison of the data with and without virus-negative samples (Additional file 1: Figure S4 and S5). This mainly resulted in changes to the first time point, but the resultant sample size was too small to reliably make any assessment.

### PRV infection in sockeye appears to show metabolic changes consistent with pathology observed in other Pacific salmon species

Infectious disease manifests on a spectrum of severity, which results from the interaction of many variables. To fully understand the range of disease impacts, a variety of epidemiological and laboratory techniques must therefore be employed. As highlighted by Polinski et al., PRV exposure affected hematocrit, and increasing loads of PRV were negatively correlated with hemoglobin concentration. Considering that various lineages of PRV are thought to lead to lysis of blood cells in other Pacific salmon species [2–6] (Supplementary text 3), we believe the most parsimonious interpretation of observations by Polinski et al. is that PRV infection leads to a comparable pathology in sockeye salmon: blood cells rupture (hemolysis), leading to reduced hematocrit and hemoglobin concentration. This mechanism would also explain the apparent PRV-induced changes to other physiological measures, such as SMR, EPOC, and EPOCdur. Hence, despite the low power of their experimental design, the data shared by Polinski et al. suggest that PRV infection in sockeye salmon may actually result in disease, i.e., a harmful deviation from the normal functional state of the host.

### Laboratory challenge results need to be situated within an ecological framework

Laboratory studies represent one important source of evidence to study disease in an ecological context, but they can also suffer serious shortcomings in the study of chronic — yet impactful — infectious disease. The ecological factors that a salmon faces in its life are too complex to replicate in the laboratory. While common practice, extrapolating laboratory-collected data to the field is speculative and needs to be approached with caution. Pairing multiple sources of data from laboratory and field studies offers the most robust approach to studying disease. Laboratory studies cannot capture external factors that may act in combination with disease to decrease survival (Additional file 1: Supplementary text 4). A minor effect in a laboratory setting may not seem biologically significant. When expanded across a whole population, however, and in combination with other cumulative effects, such small differences can have substantial impacts. Even covert infectious diseases that cause no overt symptoms can influence behavior and impact survival [14], and minor changes in behavior could result in increased predation of infected individuals [15]. Additionally, less virulent viruses can maintain a transmission advantage over agents which cause acute disease [16], resulting in higher infection prevalence, and population-level impact, despite lower severity.

In particular, we suggest that the relationship between PRV infection and EPOCdur could readily alter predator-prey dynamics for juvenile sockeye salmon. Polinski et al. concluded that their detected energetic cost of PRV infection would only manifest at “maximal exercise” and would confer only a “limited life-history-associated aerobic performance risk.” Yet, through predator interactions, wild fish are likely subject to regular, iterated bouts of aerobic “exercise” where slight reductions in performance could have lethal consequences. The early marine phase is a period of substantial mortality in salmon life history, where environmental conditions, predation, and food limitation are likely important determinants of survival [17–19]. Chronic infections, such as PRV, may have a substantial indirect influence over net predation rates [15, 20]. Mere “minor” physiological or behavioral changes (e.g. delay recovering from aerobic exertion) stand to amplify background rates of mortality. Rather than acknowledging the observed physiological effects of PRV exposure and associated potential survival impacts, Polinski et al. dismiss their findings as inconsequential. We caution that the physiological effects of PRV exposure and the associated consequences of infection in their data may well impact the survival of wild sockeye salmon.

## Conclusions

As with previous challenge studies published by some of the same authors [21, 22], the authors of Polinski et al. interpret their laboratory findings as evidence that PRV infection is of little ecological consequence, in this case for sockeye salmon. They go as far as to suggest that “a combination of host tolerance and low viral virulence has the potential to be a commensal rather than parasitic relationship.” In reality, understanding infectious disease impacts on wild fish populations requires more context regarding relevant pathogens, host responses thereto, and exacerbating ecological conditions (i.e. disease is a function of the host, the agent, and the environment). These factors combine and interact to impact (or not) fish individually and at the population level [20]. Physiological assessments in a controlled environment do not reflect the full complexities of disease manifestation, and cannot be solely relied on to assess infection risk at a population level. The potential impact of any given infectious agent needs to be considered in the relevant holistic context.

While we agree with Polinski et al. that PRV infection will not always lead to disease, the absence of acute disease in a single underpowered study, conducted with hosts buffered from environmental stress, is not conclusive — or even reliable — evidence that a virus is benign. Stating that PRV infection exhibits a “commensal” relationship with sockeye hosts stands in stark contrast to findings from the wider literature that suggest PRV (including the relevant PRV-1a lineage) is a disease-causing agent [2, 23, 24]. Although disease in one species does not guarantee disease in another, any apparent discrepancy bears much more careful consideration and experimentation. The observations in this study, which indicate that PRV infection impacts the blood cells of sockeye, are consistent with observations in other Pacific species, and thus a cause for concern. Such a finding is particularly pertinent considering the growing body of evidence that links PRV with disease [2, 25] and reduced population-level survival [26] in Chinook salmon.

The question of whether PRV infection in sockeye salmon causes a disease that impacts population productivity is highly relevant to policy makers in British Columbia, Canada. This is especially true for the management of salmon farms, which present a disease-transmission risk to wild salmon [27–31]. Despite the contradictory evidence we highlight, Polinski et al. concluded that there were no major effects of PRV on the respiratory performance of sockeye salmon, and — via a university media release — they communicated to the public that “PRV poses a very low risk to British Columbia’s population of wild Pacific salmon” [32]. Fraser River sockeye productivity has declined drastically over the past three decades [33]. Clarity on whether PRV can cause disease in sockeye is needed to determine if salmon farming operations, which play a role in the transmission of the virus [27], contribute to observed declines. Polinski et al. received “collaborative support” from the salmon farming industry, focused on a single population of one (of five) species of wild salmon in the North East Pacific, and overlooked flaws which we identified in our reanalysis. Consequently, their study should not be interpreted, as it was in the news release [32], as evidence of no impact of an aquaculture-associated virus on myriad populations of *all* Pacific salmon species. Until there is reliable evidence that PRV has no effect on sockeye, we suggest that the most appropriate management response would likely be to proceed in a precautionary manner, in accordance with Canadian federal policy [34].

## Methods

Data was obtained from the Supplementary Information of Polinski et al. (which is named Additional file 2 in their paper). Their data was uploaded as an excel file. We converted the data from the ‘Sample inventory’ tab in the excel file to a csv file (Additional file 2: Polinski_data.csv). To aid in reproducibility, our reproducible code (polinski ANOVA analysis Feb.Rmd, Polinski_plots_for_paper_Jan2021.R) and data files required to run the code (Powercorrdat.csv, dflong.csv) are provided in our Additional file 2.

### Power analysis

To assess the power of the individual pairwise comparisons of each treatment against the control, we revised the power calculations of Polinski et al., focusing on these multiple comparison tests, and calculated the power with and without Dunnett’s correction factor using the R function, pt, and critical values obtained from an online resource [35].

Additionally, we repeated the analysis incorporating a tank effect, whose standard deviation was estimated by fitting a mixed-effects model to Polinski et al.’s data. Calculations were conducted using the R functions qt and pt, along with simulations to check on the accuracy of the theoretical calculations. To reassess the power when results were subject to tank effects, we made the following simplifying assumptions: (i) at each time point there were four fish sampled from each tank, with two tanks for each of the virus treatments and control groups; (ii) the individual observations within each tank were independently, normally distributed with a common standard deviation; and (iii) there were also random tank effects that were independently, normally distributed with mean 0 and a common standard deviation. We used two different values for the standard deviation of this random factor, the minimum possible value of 0 and a moderate value of 0.5, which was substantially below our fitted estimates for SMR, EPOC, and EPOCdur (between 0.71 and 1.59). These assumptions allowed us to use the non-central *t*-distribution to calculate the power of the individual multiple comparisons [36].

All code and data files are available in the supplementary materials.

### Statistical analysis

As described by Polinski et al., certain variables (T0.5ṀO2max, T0.8ṀO2max, and AOD) were log-transformed prior to analysis. Since these variables contain 0 values, and log 0 is undefined, 0.001 was added to all of these values. Note: we are not endorsing this approach, but recreating it for consistency with the original analyses. Additionally, we applied arcsine transformation to the hematocrit values. This transformation is not described in the methods of Polinski et al. but is described within the legend of their Fig. 1.

To replicate the significant effect of PRV exposure on hematocrit at 4 weeks post treatment, reported by Polinski et al., we were forced to remove an “outlier” from the control group at that time point (ID 66). In the supplementary materials associated with the original paper, this individual was described as being visually anemic, and we believe the authors also removed this sample since it is not visible on their plots, although this is not mentioned in their paper.

We aimed to replicate the statistical analysis described by Polinski et al, in which the data were assessed by 2-way ANOVA followed by Dunnett’s multiple comparison tests “in a time-point-specific manner” (Additional file 1: Supplementary Table 1). The description of the statistical analyses in Polinski et al. is not overly detailed. Additionally, the authors used graphical user interface statistical software (pers. Comm. Mark Polinski). Therefore, we can only speculate on the specifics of the multiple comparison tests. Based on the “time-point-specific manner” description, we assume that familywise *p*-value adjustments using Dunnett’s test were conducted in groups of two contrasts between each of the two viral treatments and the shared control, ignoring the main ANOVA results.

To investigate the correlation between PRV load and each physiological response measure, we calculated the Spearman Rank Correlation (i.e., Spearman’s Rho [*ρ*]) and the cor.test function in R (Figure S1).

## Supplementary Information

**Additional file 1: Supplementary text 1.** Potential effects of PRV are disregarded, and other statistical discrepancies. **Figure S1.** Correlation of each measure and PRV copy number. **Supplementary Table 1.** Significant 2-way ANOVA results in post hoc analysis of data in a ‘time-specific manner’. **Figure S2.** The effect of tank on PRV and IHNV challenge on the IRAP and blood measures at each individual time point. **Supplementary text 2A.** Much lower than reported statistical power. **Supplementary Table 2.** Estimated statistical power for testing for treatment-vs-control differences in a model with potential tank effects. **Supplementary text 2B.** The inclusion of virus-negative fish in the viral treatment group. **Figure S3.** Ct values for all the A) PRV and B) IHNV exposed fish at all three time points. **Figure S4.** The effect of PRV and IHNV challenge on the IRAP and blood measures showing all time points merged. **Figure S5.** The effect of PRV and IHNV challenge on the IRAP and blood measures at each individual time point. **Supplementary text 3.** PRV infection in sockeye appears to result in metabolic changes consistent with PRV induced disease pathways observed in other species of Pacific salmon. **Supplementary text 4.** Suitability of laboratory studies and physiology to determine the risk posed to wild salmon.

**Additional file 2.** Contains the reproducible code and data files required to run the code.

## Data Availability

We include all code for the statistical analysis and plotting, performed using R [37], in the supplementary materials. All data was obtained from the Supplementary Information of Polinski et al.
