# Supplementary material for: Assessing the role of Piscine orthoreovirus in disease and the associated risk for wild Pacific salmon
Source: BMC Biol. 2023 May 19;21:114. doi: 10.1186/s12915-023-01548-8 (PMC10199614; doi:10.1186/s12915-023-01548-8)
Supplement: Supplementary file 1 — Additional file 1: Supplementary text 1. Potential effects of PRV are disregarded, and other statistical discrepancies. Figure S1. Correlation of each measure and PRV copy number. Supplementary Table 1. Significant 2-way ANOVA results in post hoc analysis of data in a ‘time-specific manner’. Figure S2. The effect of tank on PRV and IHNV challenge on the IRAP and blood measures at each individual time point. Supplementary text 2A. Much lower than reported statistical power. Supplementary Table 2. Estimated statistical power for testing for treatment-vs-control differences in a model with potential tank effects. Supplementary text 2B. The inclusion of virus-negative fish in the viral treatment group. Figure S3. Ct values for all the A) PRV and B) IHNV exposed fish at all three time points. Figure S4. The effect of PRV and IHNV challenge on the IRAP and blood measures showing all time points merged. Figure S5. The effect of PRV and IHNV challenge on the IRAP and blood measures at each individual time point. Supplementary text 3. PRV infection in sockeye appears to result in metabolic changes consistent with PRV induced disease pathways observed in other species of Pacific salmon. Supplementary text 4. Suitability of laboratory studies and physiology to determine the risk posed to wild salmon. [file 12915_2023_1548_MOESM1_ESM.pdf]

Supplementary Materials

Supplementary text 1: Potential effects of PRV are disregarded, and other statistical discrepancies

Our attempts to reproduce the statistical analysis in Polinski et al. revealed several discrepancies. Although we attempted to replicate the authors' analyses as closely as possible, some of the methodological descriptions were superficial enough to hinder reproduction. To aid reproducibility, we include the code used for our re-analysis as a supplementary file. With our 'best-guess' reproduction of their analysis we found a significant effect of viral treatment on EPOC in week 1 for both IHNV and PRV (Supplementary Table 1), an effect not identified by Polinski et al. We did not find mention of any specific manipulation or transformation of the EPOC data that would have precluded this relatively strong result. Upon investigation of their Figure S1, it appears that two points were removed from the control group at week 1. Excess post-exercise oxygen consumption (EPOC) is a measure of a fish's capability to recover from exhaustion while EPOC duration (EPOCdur) is the time required for this recovery. Although Polinski et al. do identify a significant increase in EPOCdur in the PRV-exposed fish, they discount this as only having a temporary effect.

The other significant contrasts that we identified using the 'time specific approach' were consistent with the manuscript (Supplementary Table 1) with the exception of the p-value for the reduced hematocrit at week 4 post challenge of PRV. This is reported as $p < 0.05$ in their Figure 1b , as p=0.02 in the text, and as $p < 0.01$ in their supplementary figure S4. We also find a significant difference when we repeat this analysis (Supplementary Table 1, p= 0.0085).

One of the most striking results of the data published by Polinski et al are differences in the standard metabolic rate SMR between the virus exposed and control groups (Figure 1). The SMR is a measure of the baseline respiration needs of the organism. Indeed - the impact of IHNV infection on SMR is discussed in depth by Polinski et al., but the relationship between PRV and SMR is not described in the main text of the manuscript despite the fact that the effect of virus infection on SMR was similar for both viruses. The lower SMR in PRV-exposed fish is evident at all time points (Figure 1C), but not significant, likely attributable to the low power of the study design.

Polinski et al. describe the correlation between SMR and viral load for IHNV (Figure 2E in their manuscript). They show that many of the IHNV-exposed samples were outside of the expected values for the SMR compared to the controls. A similar observation is made for PRV (Figure 1A), in which 11 out of 18 PRV positive samples are outside of the expected range (i.e. outside one standard deviation from the mean of the control values). Of these 11, all were in the later two time points. However, we also caution that 4 of the 6 PRV-exposed but negative fish are also outside this range and that the correlation with PRV load and SMR was not significant. Polinski et al. consider samples which are less than or greater than one standard deviation of the mean control value to be 'biologically relevant'. Under this criterion, PRV-exposure appears to cause a 'biologically relevant' reduction in SMR. Whether or not this threshold of 'biological relevance' is justified in this context requires further study. Polinski et al. inferred that a high

SMR was a result of severe infection, while fish with lower IHNV loads and also lower SMR are an adaptation to protect against hypoxia while fighting systemic infections. Here we show that PRV exposure also appears to result in lower SMR (Figure 1), which we suggest is a similar adaptation, in which PRV infected fish lower their metabolism to tolerate infection and protect against low oxygen.

The significant correlations of PRV load with hemoglobin, hematocrit and EPOCdur add weight to the evidence that PRV itself is likely responsible for these changes, rather than the ‘factors unidentified in this study’ which Polinski et al. suggest. The tendency to attribute the potential impact of PRV infection on other factors is familiar; previous studies by some of these authors found differences between control and PRV-challenged fish (both sockeye and Chinook salmon), but put them down to ‘foreign antigens’ or ‘some other agent’ [1]. We note that Polinski et al. purified PRV (from other potential infectious agents) via an ultracentrifugation gradient, making these specific ‘other factors’’ less likely.

In their analysis of variance, Polinski et al do not consider the potential existence of a tank effect. Their study was not designed in such a way that it could detect the occurrence of an external factor not related to the treatments (e.g. bacterial infection, plumbing mishap, handling-induced stress). Since there were few replicative units (2-3 tanks per treatment), there is too little data to parse out an effect of the treatment from the effect of one of these other potential factors. To explore if this was an issue, we visualized the data by treatment, tank and time point (Figure S2). Evidence of a tank effect is not uncommon. For example, EPOC and EPOCdur vary considerably between the two tanks of PRV-treated fish at weeks 1 and 4, and the response variable often varies dramatically between the two control tanks (e.g., MO2max, SMR, RMR, O2crit, ILOS, SOD, FSOD, AOD, pH). Since there are effectively only two experimental units (i.e. tanks) per treatment, more replication is needed to resolve, with any confidence, if there are truly any differences between treatments. A more satisfactory statistical analysis would account for tank effect either by using a nested ANOVA or a multilevel (mixed) model.

Other miscellaneous issues:
Whilst re-analysing the data we came across a variety of other inconsistencies, listed below:
- Their Figure 1a shows PRV load calculated for week 1 with a mean of $9.16x10^3$. This is calculated just with the PCR positive fish. Unlike most of the other plots, negative fish were not included in this plot. As far as we can tell, the mean shown at time point 0 is not based on any actual measurements.
-We are unable to repeat the same standard deviation from the mean values as their Figure 1E. We calculate a mean of 1.287 and a standard deviation of 0.207.
-Their Figure 1E is missing a data point y=1.32, x=0. Their Figure 1F is missing a data point at y=21, x=0. We were unable to calculate the same spearman rank correlation for these plots.
-Their Figure 2E is plotted with both the O2crit and the SMR on different axes. It shows the standard deviation only for the O2crit, not the SMR. It is not clear why the so-called ‘biological relevance’ interval from one measure is applied to another in this plot. Furthermore, there are only 16 data points for each measure, which appear to be just the samples from the first time point. It is not clear why.

-Their Figure 1D is also missing a data point at x=0, y=114.45.

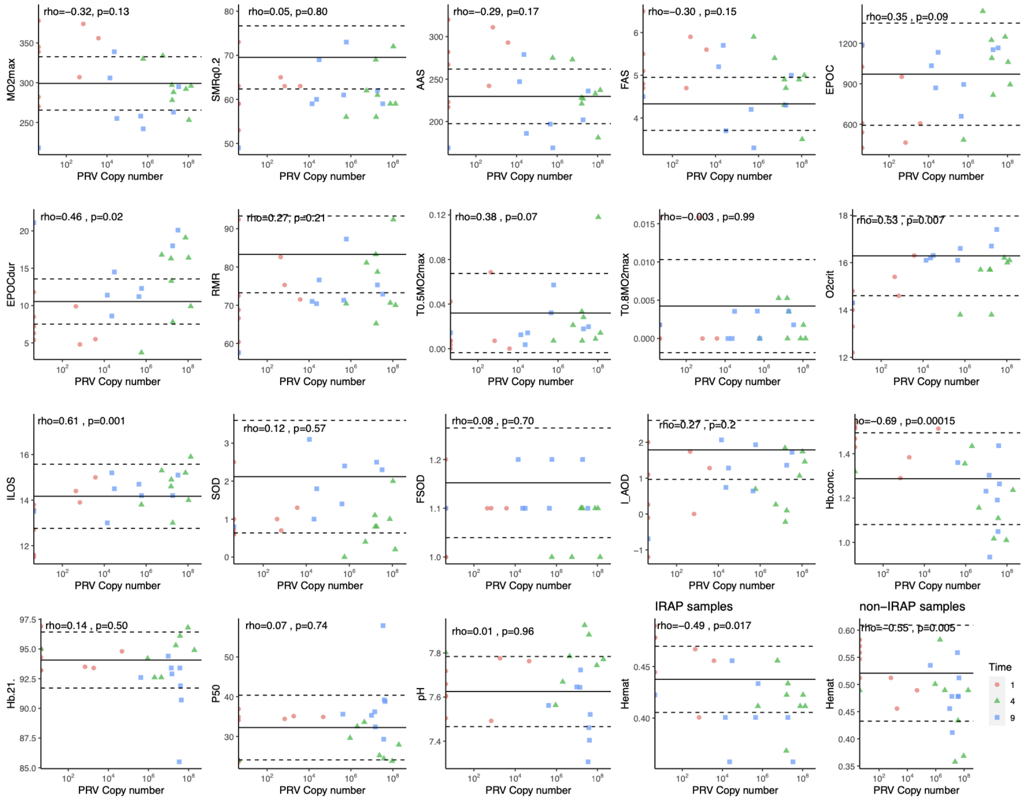

Figure S1 Correlation of each measure and PRV copy number. Horizontal solid line shows the mean of the control samples, dashed lines show the standard deviation from the mean. The spearman correlation (rho) and associated p-value are shown at the top of each plot. The key for the symbols is provided in the bottom-right plot. MO_{2max} = maximum metabolic rate; SMRq02 = standard metabolic rate; AAS = absolute aerobic scope; FAS = factorial aerobic scope; EPOC = excess post-exercise oxygen consumption; $EPOC_{dur}$ = EPOC duration; RMR = routine metabolic rate; $T0.5_{\dot{M}O2max}$ = time spent above 50% of MO_{2max}; $T0.8_{\dot{M}O2max}$ = time spent above 80% MO_{2max}; O_{2crit} = critical oxygen level, ILOS = incipient lethal oxygen saturation; SOD = scope of oxygen deficit; FSOD = factorial SOD; AOD = accumulated oxygen deficit, Hbconc. = haemoglobin concentration (mM), Hb21 = percent haemoglobin saturation at 21% oxygen, P50 = Blood partial pressure at 50% oxygen saturation (mmHg), pH = plasma pH, Hemat = hematocrit (%) , IRAP= integrated respiratory assessment protocol” (IRAP).

Supplementary Table 1 Significant 2-way ANOVA results in post hoc analysis of data in a 'time-specific manner'

Virus	Variable	Contrast	Difference between mean values	p-value 'time-specific' (to 4 decimal places)	Polinski p value (* p < 0.05; ** p < 0.01; *** p < 0.001)	Consistent with Polinski et al.	p-value if tank effect is considered
IHNV	Standard metabolic rate (SMR)	Control vs IHNV week 9	9.375	0.0457	*	Yes	0.075
IHNV	Excess post-exercise oxygen consumption (EPOC)	Control vs IHNV week 1	463.875	0.0005		No	0.0242
IHNV	Accumulated oxygen deficit (AOD)	Control vs IHNV week 1	2.0741	0.0069	*	Inconsistent p-value range (i.e. should be **)	0.0144
IHNV	Hematocrit (IRAP samples)	Control vs IHNV week 4	0.0515	0.0129	*	Yes	0.1085
IHNV	Hematocrit (IRAP samples)	Control vs IHNV week 9	0.0489	0.0147	*	Yes	0.1215
IHNV	Hematocrit (non-IRAP samples)	Control vs IHNV week 1	0.0786	0.0014	**	Yes	0.0239
PRV	EPOC	Control vs PRV week 1	463.75	0.0025		This result was not originally reported, apparently due to two control samples being removed, an error which resulted in an author correction.	0.0347
PRV	Excess post-exercise oxygen consumption duration (EPOCdur)	Control vs PRV week 9	-4.4	0.0494	*	Yes	0.0602
PRV	Hematocrit (non-IRAP samples)	Control vs PRV week 4	0.0778 (with sample 66 removed)	0.0085 (with sample 66 removed)		Yes, if we remove sample 66.	0.6370

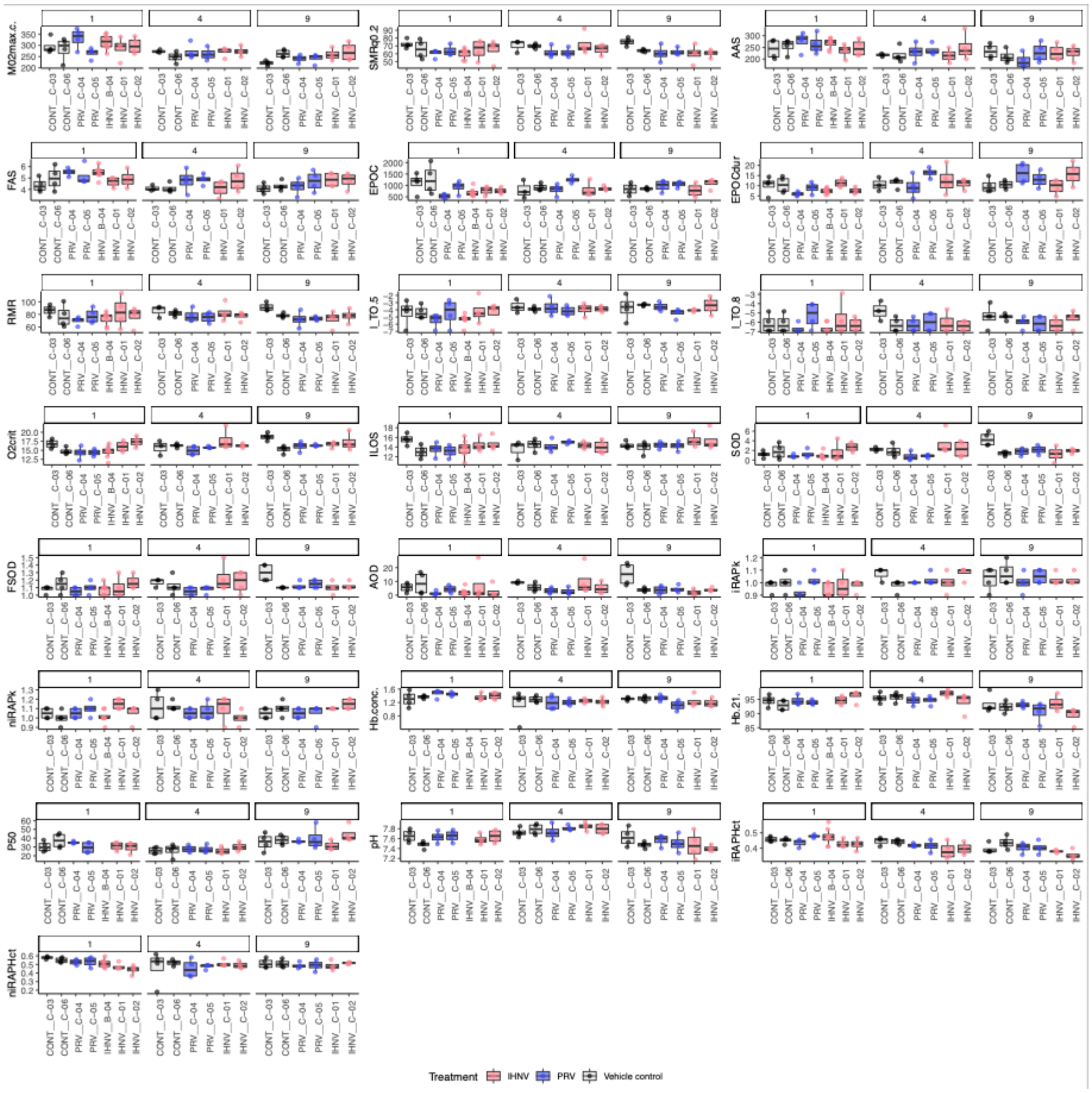

Figure S2 The effect of tank on PRV and IHNV challenge on the IRAP and blood measures at each individual time point.

Supplementary text 2A: Much lower than reported statistical power
If an experiment is conducted with too few experimental units, it may suffer a low probability of detecting even a substantial real treatment effect; i.e. the experiment may have low statistical power. We argue that Polinski et al. fundamentally misrepresented the actual power of the tests they used to make their various points.

Firstly, Polinski et al. reported power for the wrong statistical tests. The primary focus of the analyses by Polinski et al. was on their reported Dunnett's multiple comparison tests, which the authors used to compare eight (in most cases) experimentally treated fish to eight common control fish for each of IHNV and PRV treatments. Polinski et al., however, reported power calculations for the two-way ANOVAs associated with those multiple comparisons. At no point did Polinski et al. refer to the power of the multiple comparison tests that they actually discussed or to the results of the ANOVA tests for which they reported power. In the case of calculating power for multiple comparison testing in ANOVA, an extra correction factor is required to protect against spurious conclusions of significance for each individual comparison. This extra protection has the effect of lowering the power for each individual comparison, relative to the overarching ANOVA.

Secondly, Polinski et al. appear to have inappropriately inflated the power estimate they did report. In attempting to recreate the analysis of Polinski et al., the only way we could generate a power estimate for their two-way ANOVA approaching their reported value of 0.85 was if we assessed only the power of the main treatment effects, completely ignoring the possibility of interactions. Main treatment effects in this context are, by definition, constant over time. Time-specific treatment effects must involve not only main effects, but also interactions with the time variable. We know that interactions should come into play here, since Polinski et al. base much of their arguments on the fact that the two infectious-agent treatments have different effects over time: IHNV generates a fast-acting acute infection and PRV leads to a more chronic, slow-acting infection.

Finally, by not considering tank effects, Polinski et al. ignored a known potential source of variation [2]. Prior, long-established knowledge [3] of such sources of variation behooves all authors to honestly consider them in analysis; Polinski et al. needed to include a random effect of tank in their models. With only two replicate tanks per treatment combination, however, there would have typically been only three degrees of freedom available to estimate the variance associated with this random effect (within each treatment combination, there are only two tank means for estimating both the mean and variance. This leaves only a single degree of freedom for estimating the variance from each treatment combination, for a total of three.) This much smaller number of degrees of freedom substantially reduces the statistical power of the relevant statistical comparisons. To select the appropriate effect size to assess the reduced power of individual comparisons within the appropriate random-effects model, we made an approximation using Cohen's related "large effect size" for a *t*-test (0.8), which he demonstrated to be reasonably comparable to his choice for the associated analysis of variance (**f** = 0.4) [4], and which is the same as the value of an unidentified parameter, 'f', stated by Polinski et al.

We performed these power calculations with and without Dunnett's correction factor, and with two potential values for the standard deviation of the random tank factor: the minimum possible value of 0 and a value equal to half the within-tank fish-to-fish standard deviation, a ratio well within the range of estimates based on Polinski et al's results (see methods section). We present results in Supplementary Table 2.

We found the relevant power for each time-specific comparison of a metabolic measure to be much lower than the value reported by the authors – between 0.083 and 0.137 compared to the value over 0.85 reported in the paper. We attribute this difference to a combination of three, factors: (i) the authors' focus on the power of two-way analysis of variance test in place of the multiple comparisons that were the foci of Polinski et al., (ii) an apparent focus on main effects in a two-way ANOVA rather than an appropriate model involving interaction as well as main effects, and (iii) their failure to consider tank effects and resultant failure to incorporate those effects into their power calculations.

Our results are clear: the actual power was substantially lower than reported. Incorporating interactions into the ANOVA model, which we argue remains an inappropriate power calculation given the focus on multiple comparison tests by Polinski et al. (see above), reduces power for detecting time-specific treatment effects from over 0.85 to under 0.40 (see methods section below; code included in supplementary materials). Moreover, in calculating the power of the multiple comparison tests – that were the focus of the original paper – the cumulative impact of both failing to account for Dunnett's correction factor and failing to include potential tank effects is that the power for some of the tests reported by Polinski et al. could actually have been as low as 0.083, and almost certainly below 0.137 (Supplementary Table 2).

Supplementary Table 2 Estimated statistical power for testing for treatment-vs-control differences in a model with potential tank effects. The symbol, *n* represents the number of fish tested per treatment at each time point; σ_{tank}, the standard deviation of the random tank effects; and σ_{fish}, the standard deviation for the fish-to-fish variation within a tank. In the first row, with σ_{tank} =0, there are no tank effects. The second row, with $\sigma_{tank} = 0.5\ \sigma_{fish}$, was based on actual results from the experiment as described in Supplementary text 2A. Because the authors did indeed use Dunnett's correction, we conclude that the real power was likely between 0.083 and 0.137.

**Underlying Conditions** (see methods section for clarification)	**Power (with or without Dunnett's Correction)**	
	With	Without
n = 8, σ_{tank} = 0 (no "true" tank effect, but still using a random-effects analysis)	0.137	0.206
n = 8, σ_{tank} = 0.5 σ_{fish} (with tank effect and appropriate random-effects analysis)	0.083	0.128

Supplementary text 2B: The inclusion of virus-negative fish in the viral treatment group
At multiple time points throughout the experiment, 16 individuals were sampled for each treatment (viral exposure vs saline control). These were used for either the IRAP measures (*n*=8), or for the blood measures (*n*=8). At the first time point, many of the PRV-exposed fish had no detectable virus in the blood. This result is apparent in the supplementary data provided, but was not clear in their plot (Figure 1A in their manuscript), where these points were not included. We propose these virus negative fish were in the "window" period (the time between exposure and when the agent can reliably be detected [5]). Since not all fish were positive for PRV, the true sample size for detectable infections was much smaller, most notable at the first time point (Figure S3).

Negative detections of PRV were described in the paper; "At 1 week post-challenge (wpc), 6 of 16 fish (38%) had developed a moderate systemic blood infection". For Blood O_2 carrying capacity at the first time point, measurements were taken for 8 fish, but only 3 of these were RT-PCR positive for PRV. For IRAP index measures, the other 8 fish were measured, and of these only 3 fish were positive (for a total of 6 PRV positive fish; Figure S3 A). The result is that of the PRV-exposed samples at 1-wpc (n=16), only 6 are actually positive for PRV by RT-PCR. These individuals were all exposed to PRV, but they were not all necessarily infected. Nevertheless, these virus-negative fish were included in the PRV group, and inaccurately referred to throughout the manuscript as 'PRV infected'. We recognise that these individuals may have gone on to become infected with more time, but we do not expect there to be an effect of the infection on physiological measurements with an undetectable amount of virus in the blood.

Together, these analyses suggest that the study design was insufficient to reliably find an effect of PRV exposure on the blood and physiological measures. There were too few PRV positive fish to find much of an effect - especially at the first time point (Figure S3). For IHNV, additional fish were exposed and sampled which the authors justify because "dichotomy of resistance and susceptibility was expected for IHNV exposure". There is no explanation of why smaller sample sizes were tolerated for PRV.

For interest, we have made a comparison of the data with and without virus-negative samples (Figure S4 and Figure S5). As expected, the removal of these samples mostly resulted in changes to the first time point of the PRV challenge, but since the resultant sample size is so small (*n*=3), it is difficult to reliably assess the impact of PRV infection on these infected samples.

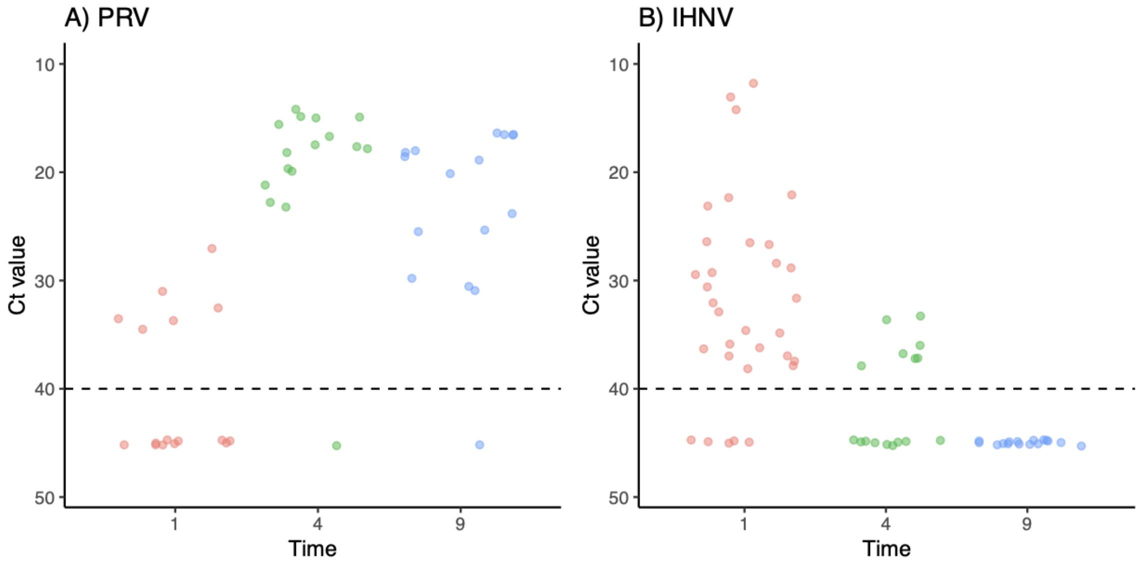

Figure S3 Ct values for all the A) PRV and B) IHNV exposed fish at all three time points; 1 (red), 4 (green) and 9 (blue) weeks post challenge. For visualization purposes, the negative samples are plotted at an arbitrary value of Ct 45. Ct values higher than 40 (shown as a horizontal dashed line) are negative, i.e. no Ct.

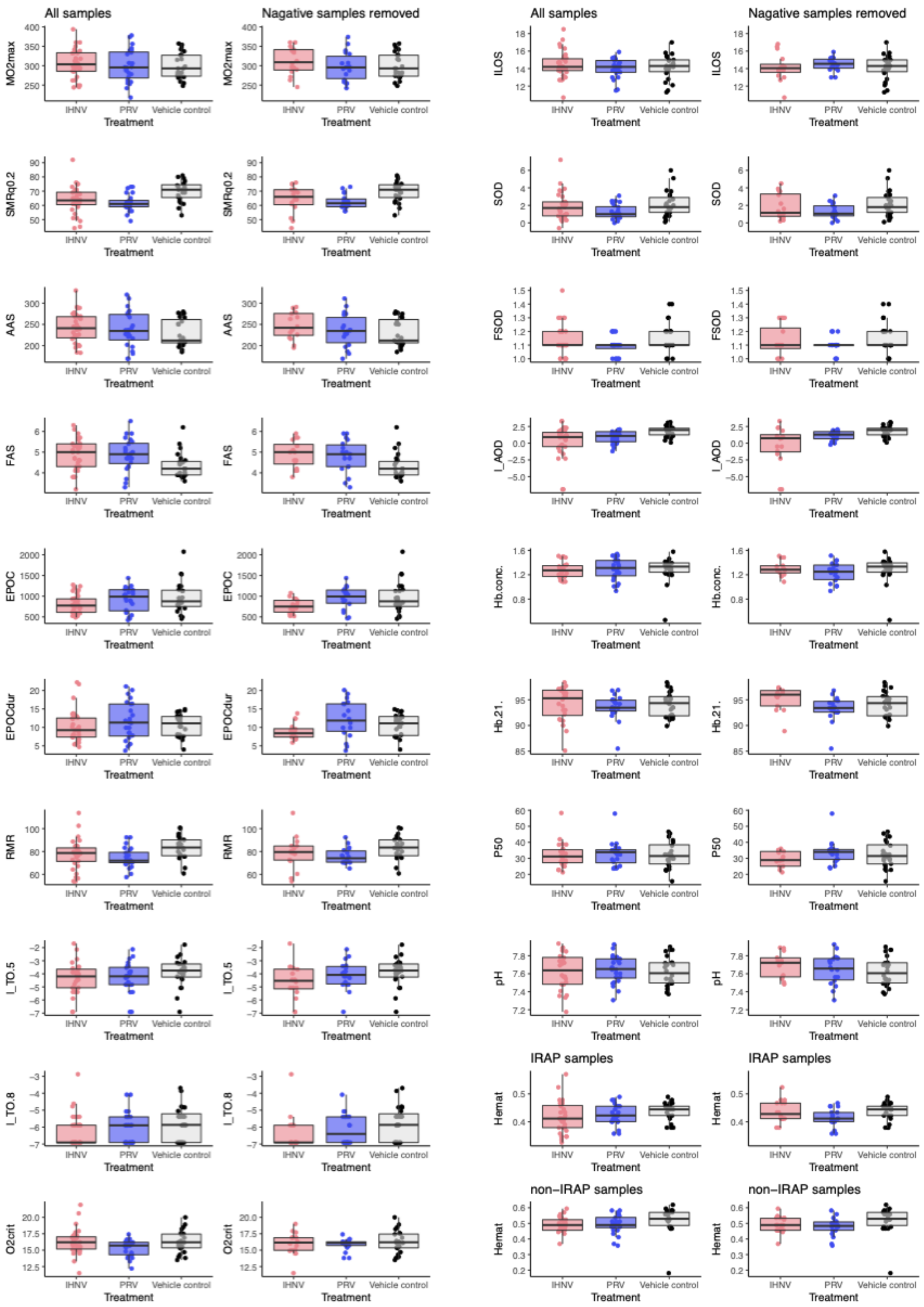

Figure S4. The effect of PRV and IHNV challenge on the IRAP and blood measures showing all time points merged. Side-by-side comparisons show the data including all samples (left of each column) and removing PRV negative samples (right of each column).

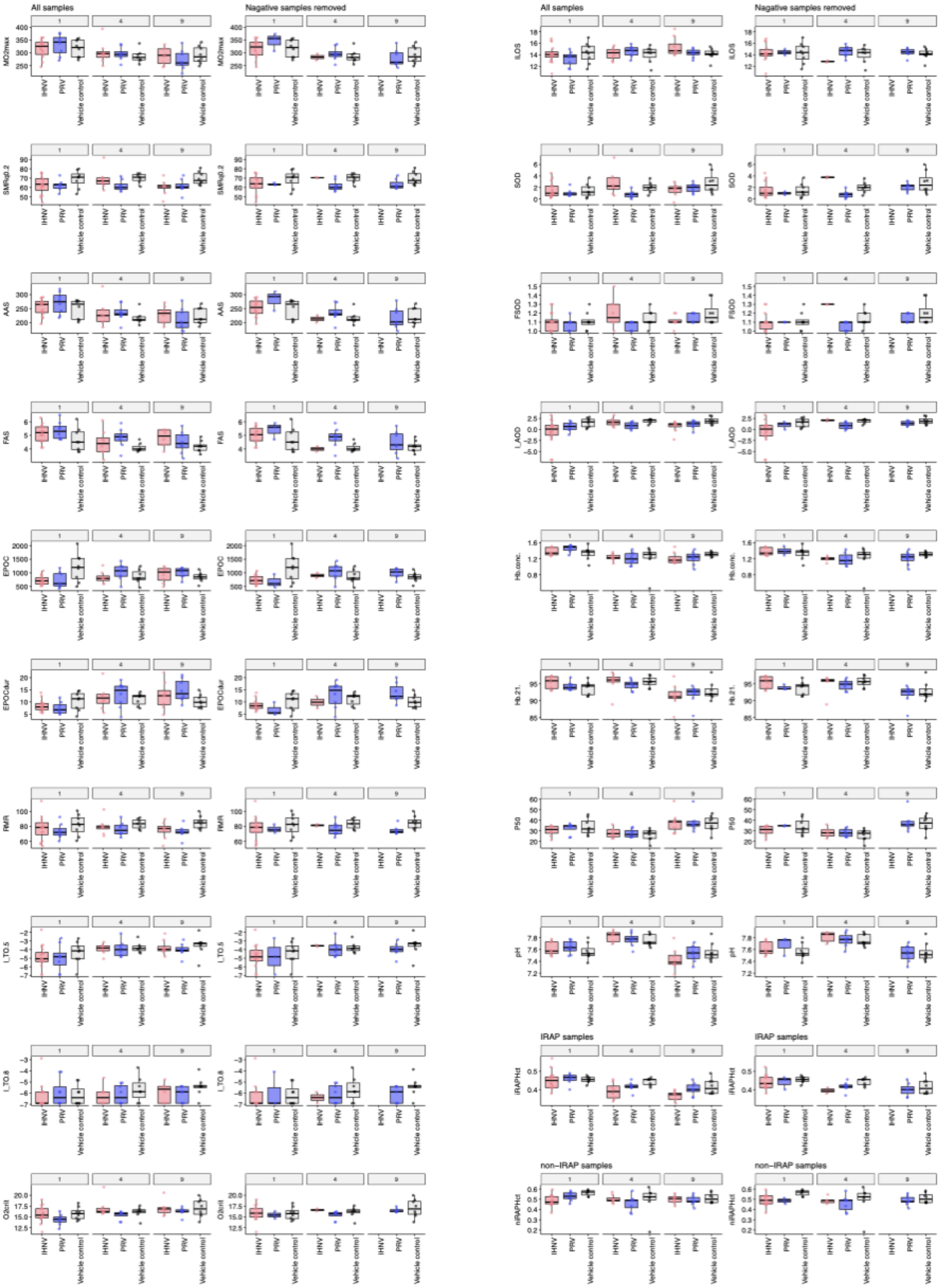

Figure S5 The effect of PRV and IHNV challenge on the IRAP and blood measures at each individual time point. Side-by-side comparisons show the data including all samples (left of each column) and removing PRV negative samples (right of each column).

Supplementary text 3: PRV infection in sockeye appears to result in metabolic changes consistent with PRV induced disease pathways observed in other species of Pacific salmon

No laboratory challenge studies have ever causally linked PRV infection with mortality [6–8] – but nevertheless, all known strains of PRV have been proven to cause disease [9–11] including the lineage of PRV-1 in BC [6]. PRV-1 has been conclusively identified as a causal agent of disease in Atlantic salmon, is epidemiologically linked with disease manifestation and mortality on Atlantic salmon farms [12, 13], and is associated with a different disease manifestation in Chinook salmon [14]. The disease pathway in Chinook salmon is consistent with PRV-related diseases in other Pacific species [9, 11, 15, 16], all of which result in substantial lysis of infected red blood cells.

Experimental evidence on the etiological role of PRV in sockeye salmon is limited and in some cases conflicting. An early challenge study in sockeye salmon found an association between PRV infection and lesions in the heart and liver [1]. However, this study was not designed to deliver causal evidence of such a link and the authors comment that these lesions could be caused by agents other than PRV. Importantly, lesions in the liver of PRV-exposed sockeye were reminiscent of those observed in experimentally exposed Chinook salmon (e.g. hepatocellular cytoplasmic iron-rich pigment granules), which are likely the result of hemoglobin accumulation and degradation after breakdown of red blood cells [1]. In Chinook salmon, excess hemoglobin impairs liver function, which in some cases results in jaundice [14]. In PRV-exposed sockeye, mild heart lesions have been observed [1], and PRV has been associated with mild to severe (but transient) heart lesions in Chinook [14]. Polinski et al. [17] reported no substantial histopathological differences between the exposed and control fish, although PRV intensity (load) was negatively correlated with blood hemoglobin concentration and hematocrit (Figure S1). We call attention to these contrasting results to highlight the fact that challenge studies that do not result in disease do not necessarily rule out the possible existence of a cause-and-effect relationship between an infectious agent and disease under different conditions. Perhaps the most famous example within the field of epidemiology is the suite of failed attempts by Cárlos Finlay (over a period of 25 years in the late 19th century) to prove his hypothesis that mosquitoes transmit yellow fever [18, 19]. Although Finlay's hypothesis ultimately proved correct, all of his attempts failed due to a flaw in his experimental setup [19].

Despite their study being severely underpowered, Polinski et al. observe that PRV exposure did lead to some physiological changes in infected sockeye salmon. Importantly, the changes they observed were in line with what one might expect in terms of disease from this particular virus. Rather than the inflammatory heart disease PRV causes in Atlantic salmon, all PRV-related diseases in Pacific salmon are the result of or induce rupture of the blood cells infected with PRV [9, 11, 14, 16, 20]. Polinski et al. observe changes consistent with this disease pathway, for example significant negative correlations between hemoglobin/ hematocrit and PRV load (Figure S1) and significantly reduced hematocrit in PRV exposed fish (Figure S5, Supplementary Table 1). If we consider the context of PRV-related diseases in other Pacific salmon species which exhibit similar pathologies [9, 11, 14, 16, 20], the most logical interpretation of these observations is that the hemolysis is mediated by PRV infection itself.

Indeed, this is presented as a potential explanation in their manuscript, although the authors speculate that host-directed removal of red blood cells might be responsible (i.e. the host initiates destruction of its own erythrocytes). Polinski et al. propose that this is a defensive tactic of the host, and we suggest that if this was the case, the reason the host would remove these cells is extrinsic i.e. because they are virus-infected and damaged. We postulate that the reduction in hemoglobin and hematocrit is a result of viral infection and eventual lysis of the blood cells (intravascular hemolysis), as well an increased turnover of PRV-infected damaged red blood cells through the reticuloendothelial system of the spleen (extravascular hemolysis). Intravascular hemolysis has been observed in other PRV-host systems [14], and extravascular hemolysis occurs as a result of viral infection in other virus-host systems including HIV [21], and Infectious salmon anaemia [22]. Regardless of the exact mechanisms of hemolysis, the limited evidence from this study supports the hypothesis that PRV infection results in lysis of red blood cells.

In wild sockeye salmon, PRV can be detected in both juveniles and adults [23], and while prevalence varies across sockeye populations it is relatively low on average [24–27]. However, there is limited sampling of sockeye in the cooler months, when PRV prevalences typically increase [12]. An exception to the low PRV prevalence is in the Columbia system; in our relatively small dataset of sockeye originating from the US (2009-2018), prevalence of PRV is 10.7% (n=139). We note that in Chinook salmon from the Columbia there is evidence of sustained transmission of a distinct sub-lineage of PRV-1a [28], and it is possible that similar transmission dynamics may be at play in sockeye originating from the same river. In an early field-based study on adult sockeye salmon returning to spawn in the Fraser River, PRV has been shown to increase the odds ratio of dying before reaching spawning grounds by 2.3 (although this result is not statistically significant) and was also significantly correlated with mortality during migration towards spawning grounds in one of two assessed stocks [23]. It is worth noting that coinfection with other agents means that we cannot be confident that PRV is responsible for this effect.

Supplementary text 4: Suitability of laboratory studies and physiology to determine the risk posed to wild salmon

Mortality in a laboratory setting is an insensitive endpoint for chronic diseases. In laboratory challenges, infection by a highly virulent virus (e.g. IHNV) leads to significant mortality, while less virulent viruses (e.g. PRV) do not. As a result, the potential risk posed by lower virulence pathogens may be disregarded, despite their etiological role and the real risk they may pose at a population level. There are numerous examples of pathogens, which in a controlled laboratory challenge would be relatively benign, but at the population level, additional factors external to the agent itself can lead to disease and/or mortality, with knock-on effects for population dynamics and persistence. For example, cowpox is endemic in British field voles, and does not cause any obvious pathology or clinical signs in either the laboratory or the field; however, it does have an impact on survival and population dynamics, which is thought to be linked to interactions with other parasites [29, 30]. Similarly, Canine parainfluenza virus 5 causes only mild to moderate respiratory illness in dogs, but more severe disease can occur with co-infection by other respiratory viruses or bacteria [31]. In wildlife, mortality rates associated with pathogens vary widely, and in most cases factors influencing disease severity are not well understood [32]. The impact of disease persistence on populations can be equal to or greater than the impacts of the initial outbreak [32], suggesting that factors external to the causative agent itself accumulate.

A variety of laboratory and epidemiological studies are required to assess the pathogenicity and potential risk a virus poses. Combining different types of studies will offer the most robust assessment (e.g. a combination of observational studies, epidemiological studies, fisheries analyses that reveal correlations with poor survival, paired release trials, laboratory challenge studies etc.) Differing results from various studies highlight how relying on just one type of evidence to make an assessment of risk is imprudent. Since PRV was only discovered in 2010 [33], there is only just over a decade worth of research on this virus. A major step forward was the demonstration that PRV-1 is the causative agent of Heart and Skeletal Muscle Inflammation (HSMI) in Atlantic salmon [10]. A later study, also in Atlantic salmon found differences in the severity of disease between viral isolates [6], with a Canadian isolate of PRV causing mild to moderate heart lesions. Meanwhile, a study in 2019 stated that there is no sustained physiological effect of the same Canadian isolate of PRV on Atlantic salmon [34]. In contrast, a study in Norway (using an isolate of PRV originating from a field outbreak of HSMI) found that infection resulted in reduced cardiac function and hypoxia tolerance [7]. Most recently, Polinski et al concluded that infection by both IHNV and PRV do not incur a major metabolic cost to sockeye salmon [17]. IHNV can cause a highly virulent, acute disease, yet exposed fish show a less pronounced change in some indices (e.g. SMR) than those infected by PRV (Figure 1). This further demonstrates that these measurements are not sensitive to assess the potential risk a virus poses to survival, since in many cases IHNV infection leads to mortality. Together, these results demonstrate that physiological assessments in a laboratory do not capture the full complexities of disease manifestation, and certainly cannot be used alone to assess risk at a population level.

For the IHNV fish, some are succumbing to disease and even mortality. The fact that there is not a pronounced aerobic cost to the individuals that survived fails to appreciate the actual impact of viral infection on the subset of fish which do not survive. In the fish which survive, or those infected with a less virulent virus such as PRV, the interferon response, and other related genetic pathways [35] may offer a more sensitive assessment of covert infection.

Physiological impairment is known to influence anti-predator performance [36], and in free-roaming animals we postulate that very minor changes in the physiology of an organism, or even small changes in behavior, could result in predation of infected individuals [37]. In nature, even seemingly harmless 'covert' infections can influence behavior and survival [38], and in some cases less virulent viruses retain a transmission advantage over acutely virulent infections [39], meaning they can end up infecting - and possibly affecting - a larger proportion of the population. Indeed, PRV infection is much more commonly detected in marine-collected wild Pacific salmon than IHNV. For these reasons, assessing the seriousness and risk of different pathogens solely based on laboratory studies is misguided. What's more, a small effect in a laboratory trial might not be significant, but extrapolated to a large population, and considering cumulative impacts, small changes in physiological responses may have a real, substantial effect on population health.

References

1. Garver KA, Marty GD, Cockburn SN, Richard J, Hawley LM, Müller A, et al. Piscine reovirus, but not Jaundice Syndrome, was transmissible to Chinook Salmon, Oncorhynchus tshawytscha (Walbaum), Sockeye Salmon, Oncorhynchus nerka (Walbaum), and Atlantic Salmon, Salmo salar L. J Fish Dis. 2016;39:117–28.

2. Kjøglum S, Grimholt U, Larsen S. Non-MHC genetic and tank effects influence disease challenge tests in Atlantic salmon (Salmo salar). Aquaculture. 2005;250:102–9.

3. Hurlbert SH. Pseudoreplication and the design of ecological field experiments. Ecol Monogr. 1984;54:187–211.

4. Cohen J. Statistical Power Analysis for the Behavioral Sciences. Routledge; 2013.

5. Konrad BP, Taylor D, Conway JM, Ogilvie GS, Coombs D. On the duration of the period between exposure to HIV and detectable infection. Epidemics. 2017;20:73–83.

6. Wessel Ø, Hansen EF, Dahle MK, Alarcon M, Vatne NA, Nyman IB, et al. Piscine Orthoreovirus-1 Isolates Differ in Their Ability to Induce Heart and Skeletal Muscle Inflammation in Atlantic Salmon (Salmo salar). Pathogens. 2020;9:1050.

7. Lund M, Krudtaa Dahle M, Timmerhaus G, Alarcon M, Powell M, Aspehaug V, et al. Hypoxia tolerance and responses to hypoxic stress during heart and skeletal muscle inflammation in Atlantic salmon (Salmo salar). PLoS One. 2017;12:e0181109.

8. Hauge H, Dahle M, Moldal T, Thoen E, Gjevre A-G, Weli S, et al. Piscine orthoreovirus can infect and shed through the intestine in experimentally challenged Atlantic salmon (Salmo salar L.). Vet Res. 2016;47:57.

9. Vendramin N, Kannimuthu D, Olsen AB, Cuenca A, Teige LH, Wessel Ø, et al. Piscine orthoreovirus subtype 3 (PRV-3) causes heart inflammation in rainbow trout (Oncorhynchus mykiss). Vet Res. 2019;50:14.

10. Wessel Ø, Braaen S, Alarcon M, Haatveit H, Roos N, Markussen T, et al. Infection with purified Piscine orthoreovirus demonstrates a causal relationship with heart and skeletal muscle inflammation in Atlantic salmon. PLoS One. 2017;12:e0183781.

11. Takano T, Nawata A, Sakai T, Matsuyama T, Ito T, Kurita J, et al. Full-Genome Sequencing and Confirmation of the Causative Agent of Erythrocytic Inclusion Body Syndrome in Coho Salmon Identifies a New Type of Piscine Orthoreovirus. PLoS One. 2016;11:e0165424.

12. Di Cicco E, Ferguson HW, Schulze AD, Kaukinen KH, Li S, Vanderstichel R, et al. Heart and skeletal muscle inflammation (HSMI) disease diagnosed on a British Columbia salmon farm through a longitudinal farm study. PLoS One. 2017;12:e0171471.

13. Løvoll M, Alarcón M, Bang Jensen B, Taksdal T, Kristoffersen AB, Tengs T. Quantification of piscine reovirus (PRV) at different stages of Atlantic salmon Salmo salar production. Dis Aquat Organ. 2012;99:7–12.

14. Di Cicco E, Ferguson HW, Kaukinen KH, Schulze AD, Li S, Tabata A, et al. The same strain of *Piscine orthoreovirus* (PRV-1) is involved in the development of different, but related, diseases in Atlantic and Pacific Salmon in British Columbia. FACETS. 2018;3:599–641.

15. Cartagena J, Tambley C, Sandino AM, Spencer E, Tello M. Detection of piscine orthoreovirus in farmed rainbow trout from Chile. Aquaculture. 2018;493:79–84.

16. Godoy MG, Kibenge MJT, Wang Y, Suarez R, Leiva C, Vallejos F, et al. First description of clinical presentation of piscine orthoreovirus (PRV) infections in salmonid aquaculture in Chile and identification of a second genotype (Genotype II) of PRV. Virol J. 2016;13:98.

17. Polinski MP, Zhang Y, Morrison PR, Marty GD, Brauner CJ, Farrell AP, et al. Innate antiviral defense demonstrates high energetic efficiency in a bony fish. BMC Biology. 2021;19.

18. Finlay C. The Mosquito Hypothetically Considered as an Agent in the Transmission of Yellow Fever Poison. Yale J Biol Med. 1937;9:589–604.

19. Clements AN, Harbach RE. History of the discovery of the mode of transmission of yellow fever virus. J Vector Ecol. 2017;42:208–22.

20. Olsen AB, Hjortaas M, Tengs T, Hellberg H, Johansen R. First Description of a New Disease in Rainbow Trout (Oncorhynchus mykiss (Walbaum)) Similar to Heart and Skeletal Muscle Inflammation (HSMI) and Detection of a Gene Sequence Related to Piscine Orthoreovirus (PRV). PLoS One. 2015;10:e0131638.

21. Sproat LO, Pantanowitz L, Lu CM, Dezube BJ. Human immunodeficiency virus-associated hemophagocytosis with iron-deficiency anemia and massive splenomegaly. Clin Infect Dis. 2003;37:e170-3.

22. Falk K, Press CM, Landsverk T, Dannevig BH. Spleen and kidney of Atlantic salmon (Salmo salar L.) show histochemical changes early in the course of experimentally induced infectious salmon anaemia (ISA). Vet Immunol Immunopathol. 1995;49:115–26.

23. Miller KM, Teffer A, Tucker S, Li S, Schulze AD, Trudel M, et al. Infectious disease, shifting climates, and opportunistic predators: cumulative factors potentially impacting wild salmon declines. Evol Appl. 2014;7:812–55.

24. Siah A, Morrison DB, Fringuelli E, Savage P, Richmond Z, Johns R, et al. Piscine Reovirus: Genomic and Molecular Phylogenetic Analysis from Farmed and Wild Salmonids Collected on the Canada/US Pacific Coast. PLoS One. 2015;10:e0141475.

25. Miller KM, Li S, Ming T, Kaukinen K, Ginther N, Patterson DA, et al. Survey of Infectious Agents Detected in Juvenile Chinook and Sockeye Salmon from British Columbia and Washington. 2017.

26. Purcell MK, Powers RL, Evered J, Kerwin J, Meyers TR, Stewart B, et al. Molecular testing of adult Pacific salmon and trout (Oncorhynchus spp.) for several RNA viruses demonstrates widespread distribution of piscine orthoreovirus in Alaska and Washington. J Fish Dis. 2018;41:347–55.

27. Morton A, Routledge R, Hrushowy S, Kibenge M, Kibenge F. The effect of exposure to farmed salmon on piscine orthoreovirus infection and fitness in wild Pacific salmon in British Columbia, Canada. PLoS One. 2017;12:e0188793.

28. Mordecai GJ, Miller KM, Bass AL, Bateman AW, Teffer AK, Caleta JM, et al. Aquaculture mediates global transmission of a viral pathogen to wild salmon. Sci Adv. 2021;7.

29. Telfer S, Lambin X, Birtles R, Beldomenico P, Burthe S, Paterson S, et al. Species interactions in a parasite community drive infection risk in a wildlife population. Science. 2010;330:243–6.

30. Burthe S, Telfer S, Begon M, Bennett M, Smith A, Lambin X. Cowpox virus infection in natural field voleMicrotus agrestispopulations: significant negative impacts on survival. Journal of Animal Ecology. 2008;77:110–9.

31. Maclachlan NJ. Fenner's Veterinary Virology Ed. 5. Elsevier Science; 2016.

32. Cassirer EF, Manlove KR, Almberg ES, Kamath PL, Cox M, Wolff P, et al. Pneumonia in bighorn sheep: Risk and resilience. J Wildl Manage. 2018;82:32–45.

33. Palacios G, Lovoll M, Tengs T, Hornig M, Hutchison S, Hui J, et al. Heart and Skeletal Muscle Inflammation of Farmed Salmon Is Associated with Infection with a Novel Reovirus. PLoS One. 2010;5.

34. Zhang Y, Polinski MP, Morrison PR, Brauner CJ, Farrell AP, Garver KA. High-Load Reovirus Infections Do Not Imply Physiological Impairment in Salmon. Front Physiol. 2019;10:114.

35. Miller KM, Günther OP, Li S, Kaukinen KH, Ming TJ. Molecular indices of viral disease development in wild migrating salmon. Conserv Physiol. 2017;5.

36. Killen SS, Reid D, Marras S, Domenici P. The interplay between aerobic metabolism and antipredator performance: vigilance is related to recovery rate after exercise. Front Physiol. 2015;6:111.

37. Furey NB, Bass AL, Miller KM, Li S, Lotto AG, Healy SJ, et al. Infected juvenile salmon can

experience increased predation during freshwater migration. R Soc Open Sci. 2021;8:201522.

38. Benaets K, Van Geystelen A, Cardoen D, De Smet L, de Graaf DC, Schoofs L, et al. Covert deformed wing virus infections have long-term deleterious effects on honeybee foraging and survival. Proc Biol Sci. 2017;284.

39. Schroeder DC, Martin SJ. Deformed wing virus: The main suspect in unexplained honeybee deaths worldwide. Virulence. 2012;3:589–91.
